# Filling the gap: Cloze probability and sentence constraint norms for 807 European Portuguese sentences

**DOI:** 10.3758/s13428-023-02196-0

**Published:** 2023-08-08

**Authors:** Sofia Frade, Andrea Santi, Ana Raposo

**Affiliations:** 1 Iscte-Instituto Universitário de Lisboa, CIS-Iscte, Lisboa, Portugal; 2 Research Center for Psychological Science, Faculdade de Psicologia, Universidade de Lisboa, Lisboa, Portugal; 3 Department of Linguistics, University College London, London, UK

**Keywords:** Cloze probability, Sentence processing, Context constraint, Prediction

## Abstract

Sentence processing is affected by the sentence context and word expectancy. To investigate sentence comprehension experimentally, it is useful to have sentence completion norms with both context constraint and word expectancy measures. In this study, two experiments were conducted to collect norms for completion of 807 European Portuguese sentences. Context constraint was measured through type-token ratio and proportion idiosyncratic responses, while word expectancy was assessed by cloze probability. Besides establishing norms for a large sample of sentences, the study investigated the impact of the production procedure and method of analysis. In Experiment 1, a single-production procedure was used, i.e., participants completed each sentence fragment with only a single response, whereas in Experiment 2, a multiple-production procedure was used, i.e., participants have to provide up to three completion words for each sentence fragment. In Experiment 2, the analyses were obtained using two distinct methods: first-response analysis and combined-response analysis. The results showed that cloze and context measures are comparable between production paradigms and that the results from both analysis methods were correlated. The advantages of each production procedure and analysis method are discussed.

## Introduction

Sentence comprehension is a critical and unique aspect of human communication. Even though sentences are constructed from a set of specific syntactic structures, we can create an almost infinite number of distinct meanings through the structures combining with a vast number of semantic items. Despite this seemingly unlimited flexibility in constructing sentences which could in theory make anticipating upcoming words or structure difficult, we frequently anticipate upcoming words when reading a book, listening to a conversation, or watching a movie. The syntactic and semantic information of the initial words of the sentence modulates the likelihood of the upcoming words. Indeed, word anticipation is a critical ability that helps comprehenders to process language information more rapidly and efficiently (Kuperberg & Jaeger, 2016, for a revision).

In the last decades, many studies have investigated the neurocognitive mechanisms underpinning word processing during sentence comprehension by manipulating word expectancy and/or sentence constraint (e.g., Hagoort et al., 2004; Kutas & Hillyard, 1980; Schwanenflugel & Shoben, 1985; Staub, 2011). Facilitation effects for processing words that are expected in a given context have been observed in lower response times in reading and naming tasks (e.g., Brothers, Swaab & Traxler; 2017; Stanovich & West, 1983), shorter fixation times (e.g., Frisson, Harvey, Drieghe, & Staub, 2017; Rayner & Well, 1996), and reduced neurophysiological responses, namely N400 amplitude reduction (e.g., Frade et al., 2021; Federmeier et al., 2007; Kutas & Hillyard, 1980) and lower activation of the left inferior frontal gyrus (e.g., Hagoort et al., 2004). The effects of word expectancy and sentence constraint have been used to inspect if sentence processing is impaired and to what extension in specific clinical populations including aphasia (e.g., Chang et al., 2016; Berndt et al., 1997), Alzheimer’s disease (e.g., Fernández et al., 2014; Nebes & Brady, 1991), and autism spectrum disorder (e.g., Pijnacker et al., 2010). In most cases, studies have reported evidence for a reduced sensitivity to sentence context and word expectancy.

To conduct valid, reliable, replicable, and comparable studies on how sentential context modulates the processing of upcoming words, it is critical to have norms for sentences that measure their constraint level and word expectancy. There are datasets available for sentences in English (e.g., Arcuri et al., 2001; Block & Baldwin, 2010; Bloom & Fischler, 1980; Lahar et al., 2004; Peelle et al., 2020; Schwanenflugel, 1986), French (e.g., Robichon et al., 1996), and Spanish (e.g., McDonald & Tamariz, 2002). For European Portuguese, there is only a dataset of sentence completion norms for children (mean age = 9.19 years) and adolescent (mean age = 14.69) populations composed of 73 contexts (Pinheiro et al., 2010). Although useful, this single dataset is clearly insufficient to apply broadly to psycholinguistic and neurocognitive research on this topic within the Portuguese population. On the one hand, these norms cannot be used with adult participants, since cognitive processes that affect sentence completion, such as word knowledge and semantic memory, undergo substantial changes throughout development (e.g., Andrade & Raposo, 2021; Bjorklund, 1987; Cronin, 2002). On the other hand, the limited number of sentences (i.e., 73) restricts the type of experiments that can be conducted, since several studies (e.g., EEG) require many more stimuli per condition.

These norming datasets comprise sentence fragments, where the last word is missing, and participants are asked to complete it (i.e., the cloze task). This allows the researcher to determine which words are used to complete each sentence and across the sample of participants the probability of each completion word. In all studies, the sentences’ fragments have been presented on a written form and participants are instructed to complete it with a word that would fit that context. Yet, studies differ in the production paradigm used for data collection. Most have employed the single-production paradigm, in which each participant completes the sentence with a single response, i.e., the first word that comes to their mind (Bloom & Fischler, 1980; Peelle et al., 2020; Pinheiro et al., 2010; Taylor, 1953). Alternatively, considering that more than one word may readily come to mind, other studies have chosen a multiple-production paradigm, in which participants have to provide up to three completion words for each sentence (McDonald & Tamariz, 2002; Schwanenflugel, 1986). To the best of our knowledge, neither of the paradigms have been simultaneously studied with the same fragments, hence it remains unknown if cloze probability measures obtained in single- and multiple-production paradigms are equivalent. We will address this question in the current study.

Norming studies most commonly measure and report the word’s *cloze probability*, a measure of word expectancy. These ratings are obtained by computing the proportion of valid responses that used a specific word to complete the sentence fragment (Bloom & Fischler, 1980; Taylor, 1953). Besides cloze probability, sentence completion data allow for the calculation of sentence constraint measures, namely the type-token ratio and the proportion of idiosyncratic responses for each sentential frame. The type-token ratio, also defined as the probability of a modal response, is estimated for each given sentence fragment by the number of different words, or types, divided by the total number of completions, or tokens, generated. It reflects the contextual constraint of the sentential fragment, as it is sensitive to the variety of completion words that are supplied by the participants (McDonald & Tamariz, 2002; Schwanenflugel, 1986). The proportion of idiosyncratic responses, i.e., valid words generated by only 1 individual, is calculated for each sentence fragment by dividing the number of words provided by a single participant by the total number of completions (Pinheiro et al., 2010; Rossi et al., 2020; Schwanenflugel, 1986). This measure also relates to the sentence constraint, as a low constraint context is more open and likely to be completed with more distinct responses across participants. Although these measures evaluate distinct properties, both have been shown to correlate with the cloze probability of the most frequently used word to complete the sentence fragment. The higher the cloze probability of the most expected word the lower the type-token ratio and the proportion of idiosyncratic responses (McDonald & Tamariz, 2002; Schwanenflugel, 1986).

The present study targets three goals. The first and main goal is to create sentence completion norms for European Portuguese validated in the adult population. In total, 807 sentence fragments with varying syntactic structure and length were tested. Sentences were intuitively designed to be of varying sentence constraint and word expectancy. For each sentence fragment, we computed the cloze probability for the most expected word, the type-token ratio, and the proportion of idiosyncratic responses for each sentence fragment. The second goal was to investigate if results are consistent across production paradigms. In Experiment 1, data were collected using the single-production procedure implemented in a paper-and-pencil task and a total of 268 sentences were tested. Experiment 2 was a computer-based task which used the multiple-production procedure to assess 539 new sentences. Importantly, a sub-set of 62 sentences were presented in both experiments, which allowed us to directly compare the results of single- and multiple-production paradigms. Finally, our third goal was to examine the extent to which the way the data is analyzed in multiple-production paradigms affects the results. For that, in Experiment 2, two analyses were implemented to calculate the sentence completion measures, one only considering the first response of each participant – first-response analysis – and the other considering the total number of valid responses of each participant (maximum of three per sentence fragment) – combined-responses analysis. Previous studies that used the multiple-production procedure have only conducted the combined-responses analysis. Thus, it remains unknown if the results obtained using first-response analysis and combined-responses analysis are comparable.

## Experiment 1

### Methods

#### Participants

One hundred and fifty-five participants (mean age = 19.94, SD = 5.81) took part in the experiment. Two participants were excluded since their native language was not European Portuguese, leaving 153 participants. All participants were students from Universidade de Lisboa. They provided informed consent to the experimental procedure, which was approved by the ethics committee of Faculdade de Psicologia da Universidade de Lisboa.

#### Materials

A total of 268 sentence fragments were created by the experimenters and designed to: (1) be of varying sentence constraints, (2) yield nouns as the most likely sentence completion, and (3) have a range of syntactic structures (however, no formal manipulation of syntactic complexity was attempted). Each sentence fragment contained between six and twelve words (M = 8.54, SD = 1.30). We created longer sentences than other cloze procedure datasets (e.g., Bloom & Fischler, 1980; Pinheiro et al., 2010), since previous studies have reported that stronger effects of context are observed for sentences which have between five and ten words (Aborn et al., 1959; Block & Baldwin, 2010). The majority of sentences ended with a determiner (e.g., articles) or preposition (85% of the fragments), which constrained the grammatical gender and/or number of the supplied completion word and increased the likelihood of completing it with a noun.

The materials were divided across five booklets, each containing 53 or 54 sentence fragments. The task instructions were presented at the beginning of the booklet, indicating that participants should attentively read each sentence fragment and write down the word that first occurs to them as a likely end of that sentence. It was emphasized that they should only use one word. The order of the sentence fragments was pseudorandomized to reduce the potential effects of lexical or semantic association between a sentence and the following one.

#### Procedure

All participants were tested in the classroom and took on average 15 min to complete the booklet they received. Each participant completed only one booklet.

#### Coding of responses

A coder inserted the written responses in an Excel database. All legible responses (*n* = 8135) were registered in the dataset correcting for spelling errors. From those responses, 39 were removed, since there were semantically or syntactically invalid words in that sentence context (e.g., “sister” to complete the sentence “Mom asked him for help slicing the”).

Following the usual practice in coding the type of responses (Peelle et al., 2020; Staub et al., 2015), the coder adjusted the responses of the participants. Specifically, if participants answered with two words, the most appropriate one was selected. For instance, when an adjective and a noun was written (e.g., “red wine”) only the noun was considered (“wine”). In cases where there were both plural and singular forms of the same response across participants, these were collapsed to the more common form. In total, 40 replacements were made (approximately 0.4% of the responses). Additionally, there were eight “no responses”, for which participants did not answer with any word^1^.

### Results

**Fig. 1:**
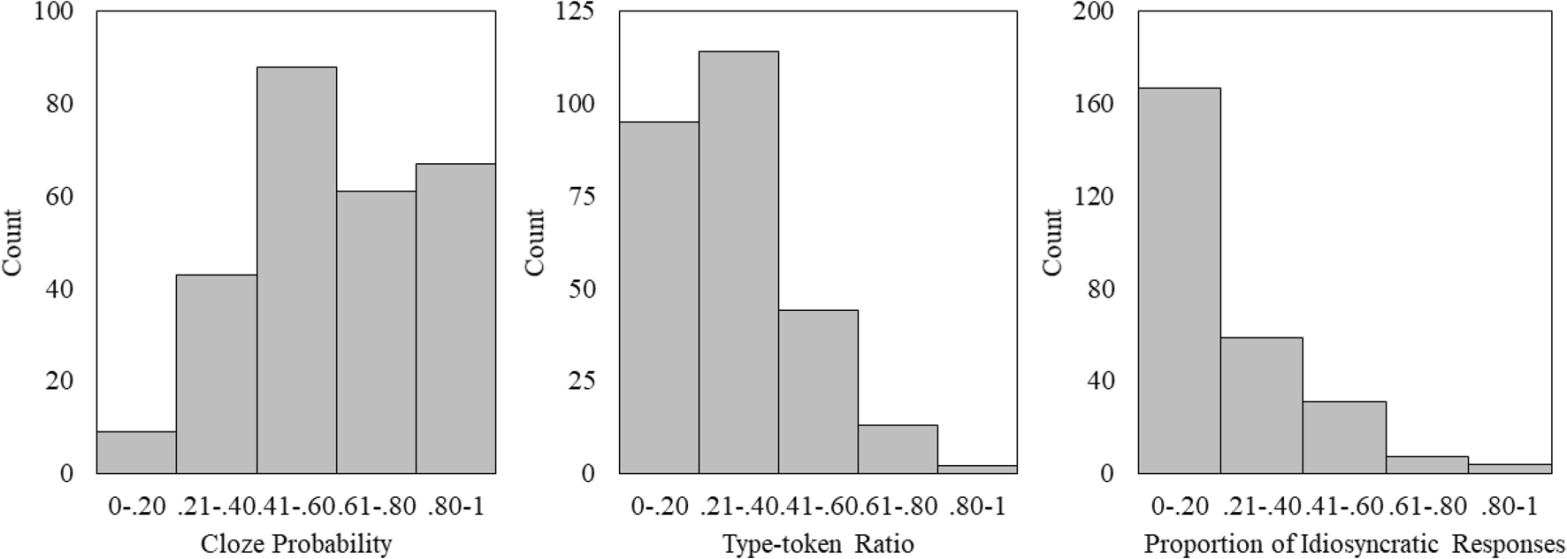
Distribution of cloze probability, type-token ratio, and proportion of idiosyncratic responses

For each sentence fragment, at least 28 valid responses were given (M = 30.23, SD =.76). Descriptive statistics for the three computed measures (cloze probability of the most expected word, type-token ratio, proportion of idiosyncratic responses) are provided in Table 1 and frequency distribution are displayed in Fig. 1. The full set of results for each sentence, which also includes the cloze probability score for all valid answers per sentence frame, is provided at https://osf.io/85xy3/.

**Table 1:** Descriptive statistics for cloze probability, type-token ratio, and proportion of idiosyncratic responses

	Mean	SE	SD	Range
Cloze probability ^a^	.60	.01	.23	.13–1
Type-token ratio	.22	.01	.13	.03–.65
Idiosyncratic responses ^b^	.11	.01	.09	0–.45

SE = standard error of mean, SD = standard deviation

^a^ Cloze probability of the most expected word. ^b^ Proportion of idiosyncratic responses

The range of cloze probability values (.13–1) reveals that the current database includes a widely varied set of sentences. The left-skewed distribution (Fig. 1) demonstrates that most sentences have a moderately to strongly expected ending word (with a cloze probability between .21 and 1). Only a few sentences have a most expected word with a very low cloze probability (< .20). This is in line with previous studies which have reported that most sentences in their datasets had a moderately to strongly expected ending word (e.g., Bloom & Fischler, 1980; McDonald & Tamariz, 2002).

Table 2 shows the Pearson correlations between cloze probability and context measures. Cloze probability was significantly correlated with type-token ratio and the proportion of idiosyncratic responses. The more strongly a word is expected in that frame, the narrower the range of completion words supplied and the smaller is the number of words only produced by one participant.

**Table 2:** Pearson correlation coefficients for cloze probability, type-token ratio, and proportion of idiosyncratic responses

	1	2	3
1. Cloze probability ^a^	–		
2. Type-token ratio	-.79^***^	–	
3. Idiosyncratic responses ^b^	-.58^***^	.91^***^	–

^a^ Cloze probability of the most expected word. ^b^ Proportion of idiosyncratic responses

^***^ *p <* .001

To further explore sentence constraint measures, the sentences were divided into five bins, split according to the cloze probability of sentences towards the most expected word (see Table 3). The mean scores of both constraint measures increased linearly across sentence bins, with higher scores of type-token ratio and proportion of idiosyncratic responses observed for sentences with lower values of cloze probability. Of note, the range of the type-token ratio and of idiosyncratic responses was large in all bins, with a clear overlap across bins. This means that some sentences have different cloze probability values but similar levels of constraint and vice-versa (i.e., cloze probability and sentence constraint are independent to some extent). This is important as it allows selecting sentences according to word expectancy or sentence constraint, as a function of the study goals.

**Table 3:** Descriptive statistics by bins of cloze probability

		TTR				IDIO			
Cloze bin	*N*	Mean	SE	SD	Range	Mean	SE	SD	Range
0.81–1	67	.09	.01	.04	.03–.21	.04	.01	.04	0–.17
0.61–0.80	61	.18	.01	.07	.07–.37	.09	.01	.07	0–.29
0.41–0.60	88	.24	.01	.09	.07–.48	.11	.01	.08	0–.42
0.21–0.40	43	.35	.02	.11	.13–.61	.20	.02	.11	0–.45
0–.20	9	.49	.03	.09	.35–.65	.26	.04	.11	.07–.41

TTR = type-token ratio, IDIO = Proportion of idiosyncratic responses, SE = standard error of mean, SD = standard deviation

## Experiment 2

### Methods

#### Participants

One hundred and sixty participants (mean age = 19.65, SD = 5.45) took part in the experiment (none of which participated in Experiment 1). They were all psychology students at Universidade de Lisboa or at Iscte-Instituto Universitário de Lisboa and received a course credit compensation for their participation. They provided informed consent to the experimental procedure, which was approved by the ethics committee of Faculdade de Psicologia da Universiade de Lisboa.

#### Materials

To increase the number of sentences with completion norms, a set of 539 new sentence fragments was created in a similar fashion to the ones used in Experiment 1. Additionally, to directly compare single- and multiple-production paradigms, we retested 62 sentence fragments from Experiment 1. In total, 601 sentence fragments were tested. Each sentence fragment contained between five and twelve words (M = 8.43, SD = 1.45).

#### Procedure

The experiment was implemented in E-Prime 2.0 experimental software (www.psnet.com) and data were collected on Windows PC computers at the faculty labs^2^. Each trial started with the presentation of a fixation cross (500 ms) in the center of the screen. Then, the sentence fragment and the response box appeared on the screen and participants had to type a word to complete the sentence. After pressing the ‘Enter’ key and a new response box appeared. The same occurred for the second word, then after the third word there was an inter-trial interval of 300 ms. Items were presented in random order and word completion was self-paced. Each participant was presented with a set of sentence fragments, ranging from 79 to 162 fragments, according to the time available to complete the task. The task duration varied between 15 and 40 min, depending on the number of sentence fragments evaluated and on participant speed.

In the beginning of the experiment, participants received instructions indicating that they should attentively read each sentence fragment and write down three words that were likely completions of those sentences. If participants could not generate three possible candidates, they were instructed to type ‘NS’.

#### Coding of responses

A coder created an Excel database with all responses. All legible responses (*n* = 35,545) were registered in the dataset after correcting for spelling errors. Three hundred and sixty-nine responses were excluded since words induced a semantic or syntactic violation of the sentence context. The same criteria for adjusting the responses used in Experiment 1 were applied in this dataset (e.g., when more than one word was used, and when responses included different forms of the same words. In total, 215 replacements were made). There were 8324 “no responses”. In 320 cases, participants have not written any word, while in the remaining cases participants have indicated (by writing ‘NS’) that they did not remember any word to complete the sentence fragment.

### Results

The mean number of participants that answered to each sentence frame was 24.85 (SD = 4.20). Participants produced an average of 2.37 words per sentence frame (SD = 0.38). All measures were computed in two ways: (1) first-response analysis – dividing the number of participants listing each response by the total number of valid responses to each item, considering only the first word produced; (2) combined-responses analysis – dividing the number of participants listing each response by the total number of valid responses to each item considering all the valid completions*.* The full set of results for both first-response and combined-responses analyses can be found at https://osf.io/85xy3/. The descriptive statistics of the computed measures (cloze probability of the most expected word, type-token ratio, proportion of idiosyncratic responses) for both analyses are displayed at Table 4.

**Table 4:** Descriptive statistics for first-response and combined-responses analyses

Analysis	Variables	Mean	SE	SD	Range
First response	Cloze probability ^a^	.58	.01	.21	.10–1
	Type-token ratio	.25	.01	.12	.03–.71
	Idiosyncratic responses ^b^	.13	.004	.10	0–.57
Combined responses	Cloze probability ^a^	.33	.004	.11	.10–.77
	Type-token ratio	.27	.003	.08	.08–.60
	Idiosyncratic responses ^b^	.15	.003	.08	0–.47

SE = standard error of mean, SD = standard deviation

^a^ Cloze probability of the most expected word. ^b^ Proportion of idiosyncratic responses

As in Experiment 1, the range of the cloze probability (see Table 4) showed that the sentences present a broad range of word expectancy of the most expected words, even though, as in Experiment 1, there are more sentences with highly compared to weakly expected words. Similar to Experiment 1, cloze probability was negatively correlated with type-token ratio and the proportion of idiosyncratic responses for both first- and combined-responses ratings (see Table 5). Notably, strong correlations were observed for the ratings of first-response analysis, while for the combined-responses ratings the correlations were weak.

**Table 5:** Pearson correlation coefficients for first-response and combined-responses analyses

Measures	1	2	3	4	5	6
1. First-response CP^*a*^	–					
2. Combined-responses CP^*a*^	.83^***^	–				
3. First-response TTR	-.76^***^	-.71^***^	–			
4. Combined-responses TTR	-.27^***^	-.29^***^	.59^***^	–		
5. First-response IDIO^*b*^	-.52^***^	-.52^***^	.90^***^	.63^***^	–	
6. Combined-responses IDIO^*b*^	-.14^***^	-.13^**^	.40^***^	.91^***^	.50^***^	–

CP = cloze probability, TTR = type-token ratio, IDIO = idiosyncratic responses

^a^ Cloze probability of the most expected word. ^b^ Proportion of idiosyncratic responses

^***^ *p <* .001; ^**^ *p <* .01

Comparison of the most frequent response across analysis (i.e., between the first response analysis and combined response analysis) showed that in 87.02% of the sentence fragments the same word appears as the most frequent response. When the most expected response was different, in most cases (64 out of 78) the most expected word in the first-response analysis was the second most expected word in the combined-responses analysis. Moreover, we found a strong correlation between the cloze probability computed using first-response analysis and combined-response analysis, whereas sentence constraint measures (type-token ratio and proportion of idiosyncratic responses) were moderately correlated between analysis (see Table 5).

Table 6 provides the descriptive statistics for type-token ratio, proportion of idiosyncratic responses and number of responses per participant for each of the five bins of cloze probability (all scores, except the number of responses, were extracted from the first-response analysis). Consistent with Experiment 1, increasing scores of type-token ratio and of proportion of idiosyncratic responses were observed for sentences with lower level of cloze probability. Though, the large range of type-token ratio and of proportion of idiosyncratic responses reveals that for sentences with similar word expectancy there are sets of sentences with differentiated levels of sentence constraint. The mean number of responses did not vary linearly across bins. The number of responses was high and very similar across bins, with the exception of the one with the highest cloze probability bin, which has a slightly lower average.

**Table 6:** Descriptive statistics by bins of cloze probability

Cloze bin	N	TTR				IDIO				Number of responses			
		Mean	SE	SD	Range	Mean	SE	SD	Range	Mean	SE	SD	Range
0.81–1	102	.11	.01	.05	.03–.24	.05	.01	.05	0–.19	2.07	.04	.40	1.29–2.86
0.61–0.80	147	.20	.01	.06	.07–.41	.10	.01	.07	0–.36	2.38	.03	.37	1.10–2.96
0.41–0.60	208	.27	.01	.09	.08–.57	.13	.01	.09	0–.48	2.45	.02	.34	1.10–3
0.21–0.40	137	.37	.01	.10	.14–.67	.20	.01	.11	0–.57	2.46	.03	.32	1.50–3
0–.20	7	.55	.06	.12	.36–.71	.30	.06	.09	.18–.43	2.58	.13	.29	2.09–2.91

TTR = type-token ratio, IDIO = Proportion of idiosyncratic responses, SE = standard error of mean, SD = standard deviation

**Fig. 2:**
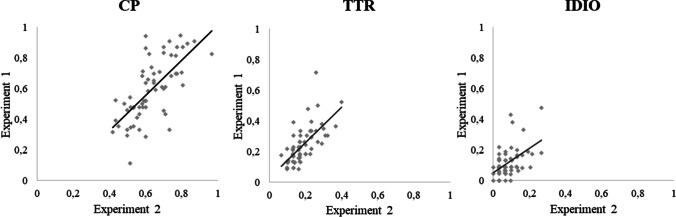
Relationship between Experiment 1 and Experiment 2

To assess the responses generated in both production paradigms, we compared a sub-set of sentences (*n* = 62) tested in both Experiments 1 and 2 using the same type of analysis, i.e., focusing on the first word produced. As shown in Table 7, the mean values of the computed measures are numerically close (changes are below .06). Yet, the distribution measures, namely in the range values, and the scatterplot (Fig. 2), show that the score computed from the multiple-production procedure had greater variability. Notably, all the computed measures were moderately correlated between experiments (cloze probability: *r* = .71, *p* < .001; type-token ratio: *r* = .697, *p* < .001; proportion of idiosyncratic responses: *r* = .530, *p* < .001). Additionally, for 52 of the 62 sentences (84%) the most expected word was the same in both procedures. Noteworthy, for the ten sentences in which the most expected word differed, in nine of those sentences, the most expected words in the single-production were the second most expected in the multiple-production paradigm.

**Table 7:** Descriptive statistics for the sub-set of sentences presented in both production paradigms

Paradigm	Variable	Mean	SE	SD	Range
Single-production	Cloze probability ^a^	.63	.02	.12	.42–.97
	Type-token ratio	.18	.01	.07	.07–.40
	Idiosyncratic responses ^b^	.08	.01	.06	0–.27
Multiple-production	Cloze probability ^a^	.59	.03	.19	.10–.91
	Type-token ratio	.24	.02	.12	.09–.71
	Idiosyncratic responses ^b^	.12	.01	.10	0–.48

SE = standard error of mean, SD = standard deviation

^a^ Cloze probability of the most expected word. ^b^ Proportion of idiosyncratic responses

## General discussion

The present study aimed to create a dataset of European Portuguese sentence completion norms to aid research on the effects of context and word expectancy in language processing. In recent years, there has been an increasing effort to validate norms for the Portuguese population, including norms for images (e.g., Garrido & Prada, 2017; Soares et al., 2014; Souza et al., 2021), sounds (e.g., Soares et al., 2013), videos (Cipriano et al., 2022), affective words (Soares et al., 2012) and emoji and emoticons (Rodrigues et al., 2018). These norms have boosted experimental research with Portuguese-speaking participants (e.g., Barriga-Paulino et al., 2022; Pereira et al., 2021; Sousa et al., 2021; Souza, et al., 2022). In language research, particularly on the sentence level, the only dataset of final word completion norms available is based on children and adolescents and a small number of items (Pinheiro et al., 2010). In our study, a total of 807 sentences were evaluated by young and healthy adults, of which 268 sentences were collected using the single-production paradigm, while 601 were collected in a multiple-production paradigm. The dataset included a wide-ranging distribution of single cloze probabilities (from .10 to 1) in both paradigms. In contrast with prior work that only tested sentences with strong contextual constraint (e.g., Block & Baldwin, 2010), our dataset contains sentences that vary in their contextual constraint. Even though we obtained a smaller number of sentences with a very low cloze probability (below .20), that does not undermine the relevance and utility of this database. Usually, studies consider weakly expected words to have a cloze probability below .45 (e.g., Federmeier et al., 2007; Thornhill & Van Petten, 2012), and as such, the present dataset provides a sufficient number of sentence frames with weakly expected words. In this dataset, the normed sentences were equally distributed into three strengths of constraint, with approximately one-third being weakly constrained (< .50 cloze probability), one third moderately constrained (cloze probability between .5 to .7) and the remaining strongly constrained (>.7 cloze probability).

The complementary measures revealed that each sentence was often completed with multiple words. In particular, each sentence fragment was completed on average with six distinct words. Most of these were evoked by more than one participant, since the mean proportion of idiosyncratic responses was lower than the type-token ratio (M = 3). These measures were correlated with cloze probability: for sentences associated with a higher cloze probability fewer words were used to complete the sentences and there was a lower proportion of idiosyncratic responses. These findings were consistent across paradigms (single vs. multiple production) and analysis methods (first- vs. combined-responses), and are in line with prior literature (McDonald & Tamariz, 2002; Schwanenflugel, 1986)*.* Critically, these measures added information regarding the contextual constraint of each sentence fragment, as they reflect the variety of completion words supplied by the participants (McDonald & Tamariz, 2002; Schwanenflugel, 1986)*.* Using that information, it is possible to differentiate the strength of context for sentences that have an equivalent cloze probability*.* For example, the sentence “*A Maria pendurou um quadro naquela*” (Maria hung the painting on that) had high cloze probability, since the most frequently word used to complete it was “*parede*” (wall) with .86 of single cloze probability, and a strong context constraint, since there was only one alternative answer “*sala*”(room); while the sentence “*O António foi à biblioteca consultar um*” (António went to the library to see a) had the same single cloze probability for the word “*livro*” (book), but had a weaker context constraint, since several alternative answers were provided by the participants “*amigo*”, “*dicionário*” e “*site*” (friend, dictionary, and site). Instead of splitting sentences in high and low constraint considering only the cloze probability, as it has been frequently done in previous studies (e.g., Federmeier et al., 2007; Ng et al., 2017; Thornhill & Van Petten, 2012)*,* it is more accurate to use one of these measures. Although, they are related with cloze probability, they provide a more precise measure of the sentence constraint, since they rely on the amount and type of words used to complete each sentential fragment.

In the multiple-production paradigm, two methods (first-response and combined-response) were used to compute the sentence completion scores. Results showed that all computed measures were strongly or moderately correlated across the two methods, which ensures that the findings are comparable when using one or the other type of analysis to select the stimulus for research. The lower values of cloze probability on the combined-response analysis was expected as the number of responses for a given word is divided by all valid answers, and thus the denominator could increase up to three times compared with the single method. Some studies have calculated the multiple cloze probability score in a different manner, by dividing the number of answers for the same word by the number of participants (McDonald & Tamariz, 2002; Schwanenflugel & Shoben, 1985). This method shifts upwards the multiple cloze probability score. However, the sum of the cloze probability of the valid answers is, in this case, different from one and for that reason we did not employ this formula. Importantly, the most frequently produced word was consistent across methods, considering only the first response or all the valid responses. Thus, data obtained using first- or combined-response analysis are closely related and are thereby comparable.

Turning to the types of production paradigms, the main advantage of using a multiple production paradigm relative to the single-production paradigm is in assessing more thoroughly the range of expected words for that sentence fragment. On one hand, participants are not required to select only one word if they have generated more than a single word to complete that sentence. On the other hand, besides the strongest candidate, there might be a second or a third strong candidate. For instance, the sentence “Every day the grandmother waters her” had one strong expected word (i.e., “plants”) when cloze probability was computed only with the first response (CP_single_ = .74) with the word “flowers” emerging as a weakly expected word (CP_single_ = .17). Yet, when computing the multiple cloze probability score, we see that the word “flowers” is produced the same number of times as the word “plants” (20 response for each out of 23 participants). This result shows that the word “flowers” is not a weakly expected word in that sentence context as it could have been assumed if only the first response was available. Therefore, selecting stimuli from a normative set that has been tested using a multiple production paradigm is more informative about the expectable candidates. This can be especially relevant if the researcher needs to select unexpected words to complete the sentence fragments, which is a commonly used condition in experimental paradigms of sentence processing (Federmeier et al., 2007; Frade et al., 2021; Thornhill & Van Petten, 2012), since it provides a more complete list of expectable words for each sentence.

The set of sentences tested in both paradigms, single and multiple production, had comparable scores, as the measures were correlated between experiments*.* Moreover, in most of the sentences the same word was produced to complete the sentence fragment. Besides the differences in the methodology, participants were also different between experiments, even though belonging to the same population, i.e., undergraduate students. The strong and significant correlations found for all scores computed in both experiments for the same items provide robust evidence for the reliability of the cloze probabilities scores. As this task relies strongly on semantic knowledge, namely on the strength of semantic associations, and on linguistic aspects, such conditional probability of a word in context, the consistency of the scores might suggest that participants have a similar semantic knowledge and linguistic exposure. Previous studies have also reported a high degree of consistency for cloze probability, especially for sentences where the final word has a high cloze probability (Block & Baldwin, 2010; Lahar et al., 2004). Our study expands this finding by revealing that sentence constraint measures also demonstrate a high degree of consistency. Comparing the same set of sentences in the two production paradigms revealed that asking for multiple words to complete each sentence fragment or only the first word that comes to mind does not change considerably the pattern of responses, although the multiple-production paradigm led to a greater degree of variability on the computed measures. This could be an indirect effect of the paradigm, since providing only a single opportunity to give a response might lead the participants to be more selective at producing their response, which may lead to more prototypical responses.

In spite of the consistency found in these experiments, caution should be taken when generalizing the norms to other Portuguese-speaking population (e.g., Brazil, Angola) or to different ages (e.g., old adults), since cloze probabilities can be influenced by the cultural and linguistic environment, as pointed out by previous literature (Arcuri et al., 2001; Bloom & Fischler, 1980; Carneiro et al., 2004; Comesaña et al., 2014)*.*

In summary, we present norms of sentence completion in European Portuguese for the young adult population. Besides the cloze probability, two additional measures – type-token ratio and proportion idiosyncratic responses – were computed to provide more information regarding the sentence context constraint. Data showed that cloze measures are robust and comparable between production paradigms and across different methods of analysis. This set of sentence-completion norms is expected to contribute to cognitive and neural research using the European Portuguese language, namely aiding in the selection and characterization of stimuli to be employed in experimental paradigms of sentence processing, and in assessing participants’ responses in such studies.

## Footnotes

1 The number of “no responses” per sentence is provided in the full dataset of this experiment.

2 Due to the suspension of the lab collection during the COVID-19 pandemic, the last participants had to perform the task remotely (36 out of 160 participants). The experiment was adapted to E-Prime Go to run the remote data collection.
